# Measurement of glomerular filtration rate in patients undergoing obesity surgery

**DOI:** 10.1186/s12882-018-1188-7

**Published:** 2018-12-29

**Authors:** Ling L. Chuah, Alexander D. Miras, Laura M. Perry, Andrew H. Frankel, David J. Towey, Zahraa Al-Mayahi, William Svensson, Carel W. le Roux

**Affiliations:** 1 Section of Investigative Medicine, Imperial College London, 6th floor Commonwealth Building, Hammersmith Hospital, Du Cane Road, London, W12 0NN UK; 2 Radiological Sciences Unit, Imperial College Healthcare NHS Trust, London, UK; 3 Nephrology, Imperial College Healthcare NHS Trust, London, UK; 4 Nuclear Medicine, Imperial College Healthcare NHS Trust, London, UK; 5 Gastro Surgical laboratory, University of Gothenburg, Gothenburg, Sweden; 6 Diabetes Complications Research Centre, Conway Institute, University College Dublin, Dublin, Ireland

**Keywords:** Renal, Creatinine, Bariatric, Cr-EDTA, GFR

## Abstract

**Background:**

Most studies on obesity surgery have measured renal function using the estimated GFR. However, due to the reduction of muscle mass, and therefore creatinine that accompanies weight loss, such measures can falsely suggest an improvement in renal function. To balance the risks of surgery versus any potential benefits on renal function, we need to be able to determine renal function using valid and reliable methodologies. In this pilot study we aimed to measure renal function in patients with CKD undergoing obesity surgery using the gold standard ^51^Cr-EDTA GFR clearance methodology which is independent of measures of muscle mass.

**Methods:**

Nine consecutive obese patients with CKD underwent obesity surgery. Their renal function was assessed using ^51^Cr-EDTA GFR, cystatin C and serum creatinine as well as using eGFR equations including MDRD CKD Epi, Cockcroft Gault and CKD Epi cystatin before and 12 months after surgery.

**Results:**

Renal function using the ^51^Cr-EDTA measured GFR did not change significantly after surgery. Similar results were obtained when Cystatin C, CKD Epi cystatin, CKD Epi cystatin creatinine and adjusted Cockcroft Gault Creatinine clearance methods were used. In contrast there were either trends or significant improvements in renal function measured using the MDRD and CKD Epi equations.

**Conclusions:**

In this pilot study using the gold standard ^51^Cr-EDTA method we found stabilisation in renal function after obesity surgery. Until further definitive data emerge it is critical to balance the risk and benefits of surgery, especially if renal function may not improve as often as previously suggested.

**Trial registration:**

ClinicalTrials.gov NCT01507350. Registered June 2011.

## Background

Obesity surgery provides significant benefits to obese patients and there is an increasing number of these operations being undertaken in obese patients with chronic kidney disease (CKD) [1]. Previous studies and metanalyses have shown that obesity surgery reduces albuminuria and proteinuria i.e. has a positive effect on renal damage, yet the effect of surgery on renal function is unknown [2, 3]. The majority of the available literature has measured renal function using the estimated GFR [Modification of Diet in Renal Disease (MDRD)/ chronic kidney disease epidemiology collaboration (CKD Epi)] [2, 4]. However, due to the reduction of muscle mass, and therefore creatinine, that accompanies weight loss after obesity surgery, such measures can be misleading as numerically calculated eGFR using the MDRD and CKD Epi equations(adjusted to BSA of 1.73m^2^) may falsely suggest an improvement in renal function [5].

Due to the association between obesity and CKD, an increasing number of patients will qualify for obesity surgery. For patients and clinicians to be able to balance the risks of surgery versus any potential benefits on renal function, we need to be able to determine renal function pre and post operatively using valid and reliable methodologies.

In this pilot study we aimed to measure renal function in patients with CKD 12 months after obesity surgery using the gold standard chromium-51 labelled ethylenediamine tetraacetic acid (^51^Cr-EDTA GFR) clearance methodology which is independent of measures of muscle mass. We also compared the results from this methodology with those from more commonly used methods.

## Methods

Nine consecutive patients with body mass index (BMI) ≥35 kg/m^2^ and eGFR (MDRD) < 60 mL/min were recruited from our obesity clinic independently of the cause of their CKD. They were eligible for bariatric surgery based on the UK NICE guidelines [6]. Their renal function was assessed using a variety of methods including ^51^Cr-EDTA GFR, cystatin C and serum creatinine as well as using eGFR equations including MDRD, CKD Epi, Cockcroft Gault and CKD Epi cystatin before and 12 months after bariatric surgery. The body surface area is calculated according to the DuBois formula [7].

^51^Cr-EDTA GFR was ascertained using bolus injection of 1.46–2.66 MBq ^51^Cr-EDTA, with between 6 and 8 venous blood samples were collected at approximately 15, 30, 60, 90, 120, 180, 240, and 300 min to assess ^51^Cr-EDTA clearance. ^51^Cr-EDTA GFR was calculated using the Bi-exponential Fitting Method described in the British Nuclear Medicine Society guidelines [8]. The prepared standard and patient samples were counted for 15 and 60 mins respectively using a Wallac 1470 Wizard Gamma Counter (Perkin Elmer Inc., Waltham, Massachusetts, USA). The measured GFR was scaled to BSA in order to maintain uniformity in comparison to reported eGFR.

Descriptive statistics were expressed as median (interquartile range) given the sample size of 9 subjects. Within groups, comparisons were made using the Wilcoxon matched pairs test given the sample size. The Pearson methodology was used to test for correlations (Graphpad PRISM software version 5.01). Statistical significance was accepted as *p* < 0.05. This was a pilot study without formal a priori power calculations.

## Results

Subject characteristics are shown in Table 1. Subjects had diabetic kidney disease (*n* = 1), non-steroidal anti-inflammatory drug induced nephropathy (*n* = 1), unilateral nephrectomy for renal cell carcinoma (*n* = 1), hypertensive nephropathy (*n* = 1), and unknown causes of CKD (*n* = 3). Two subjects had renal transplantation before obesity surgery secondary to diabetic kidney disease and focal segmental glomerulosclerosis respectively. Seven subjects had Roux-en-Y gastric bypass (RYGB), one had adjustable gastric banding, and one had a vertical sleeve gastrectomy (VSG).

**Table 1:** Pre and post-operative measurements

	Pre-op (*n* = 9)	Post-op (*n* = 9)	*P* value
Age (year)	62.0 (50.5–63.0)		
Male (%,n)	55.56%, 5		
Weight (kg)	127.0 (116.5–144.8)	100.2 (83.6–110.9)	0.004
BMI (kg/m^2^)	45.5 (42.2–47.8)	34.3 (29.3–38.3)	0.008
BSA (m^2^)	2.28 (2.18–2.45)	2.08 (1.97–2.14)	0.004
Creatinine (μmol/L)	122.5 (96.8–149.5)	110.0 (88.0–116.0)	0.01
Cystatin C(mg/L)	1.6 (1.3–2.1)	1.7 (1.4–1.8)	0.64
EDTA GFR	58.0 (48.5–91.0)	59.0 (48.9–72.5)	0.38
EDTA GFR corrected for BSA (ml/min/1.73 m^2^)	43.6 (37.0–65.0)	45.8 (41.7–63.2)	0.30
MDRD eGFR (mL/min/1.73m^2^)	51.0 (38.3–60.3)	53.5 (42.3–65.3)	0.08
CKD Epi (mL/min/1.73m^2^)	54.0 (42.0–65.0)	61.0 (48.5–71.0)	0.008
CKD Epi cystatin (mL/min/1.73m^2^)	39.0 (27.0–49.0)	40.0 (34.5–49.8)	1.0
CKD Epi cystatin creatinine (mL/min/1.73m^2^)	43.0 (`33.0–56.0)	47.5 (42.0–54.8)	0.34
Cockcroft Gauld Creatinine clearance, adjusted (mL/min/1.73 m^2^)	77.2 (61.6–89.0)	75.0 (64.1–92.3)	0.93

Abbreviations: *BMI* body mass index, *BSA* body surface area, *CKD Epi* chronic kidney disease epidemiology collaboration, *EDTA* ethylenediamine tetraacetic acid, *eGFR* estimated glomerular filtration rate, *MDRD* modification of diet in renal disease

After surgery BMI and creatinine reduced significantly. There were no major complications peri-operatively for any of the patients and none developed symptomatic kidney stones. There was no significant change in the number of blood pressure lowering medications [2.0 (1.3–2.0) vs. 2.0 (0.5–2.0), *p* = 1.0].

**Fig. 1:**
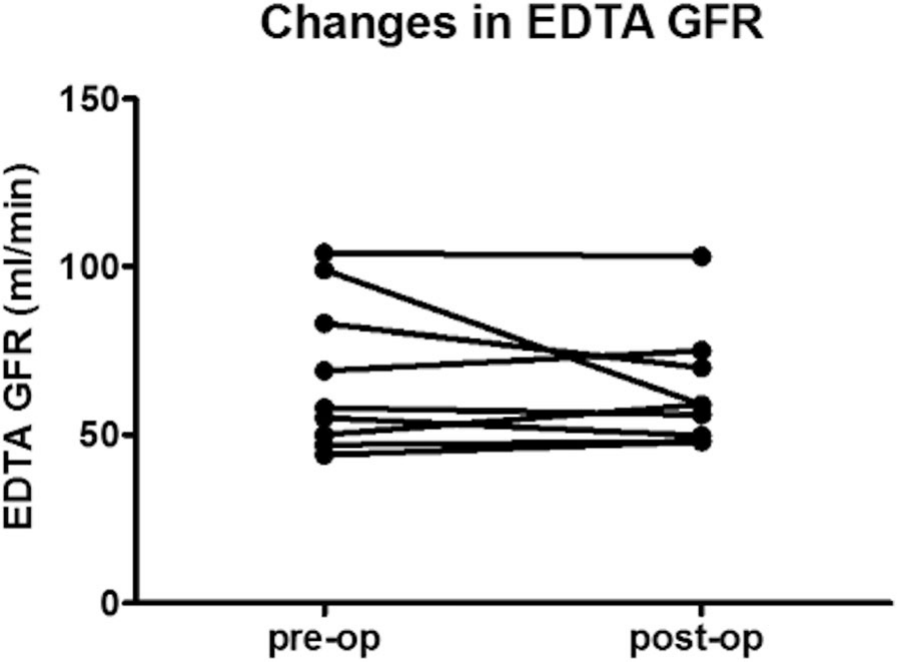
Individual ^51^Cr-EDTA GFR measurements before and after obesity surgery

Renal function using the ^51^Cr-EDTA measured GFR did not change significantly after surgery (Fig. 1). Similar results were obtained when Cystatin C, CKD Epi cystatin, CKD Epi cystatin creatinine and adjusted Cockcroft Gault Creatinine clearance methods were used (Table 1). In contrast there were either trends or significant improvements in renal function measured using the MDRD and CKD Epi equations. When the unadjusted Cockcroft Gault Creatinine clearance methodology was used, there was a reduction in renal function as the equation incorporates weight into the measure.

## Discussion

This pilot study has demonstrated that renal function as measured by the gold standard ^51^Cr-EDTA GFR method remained stable 12 months after bariatric surgery. This is in contrast to trends for or significant improvements in renal function when assessed using the MDRD equation or CKD Epi. MDRD GFR and CKD epi are known to underestimate mGFR as these methodologies are dependent on the serum creatinine, which is in turn influenced by total muscle mass. Following surgery, the reduction in weight and serum creatinine led to increase in MDRD GFR and CKD Epi, bringing it closer to mGFR.

We presented mGFR and mGFR corrected to BSA. Whilst recognised that indexation of glomerular filtration rate for BSA in obese patients could underestimate the real GFR [9], due to the reduction in significant amount of weight in obese patient post weight loss surgery; we reported the mGFR correction for BSA in order to facilitate comparison with reported eGFR corrected for BSA.

The current literature on the effects of obesity surgery on renal outcomes appears promising [4]. Surgery has been reported to reduce glomerular hyperfiltration, albuminuria and proteinuria in obese patients with or without type 2 diabetes mellitus [2]. In well matched observational cohort studies obesity surgery was associated with a 58% lower risk of eGFR decline of ≥30 and 57% lower risk of serum creatinine decline or development of end-stage renal disease in patients with and without CKD [10]. However, these findings need to be interpreted with caution as GFR was estimated and not actually measured. Within our small study group, stabilisation of renal function was found; which is a positive and encouraging finding is in itself.

Reassuringly our findings are consistent with another study that assessed 12 patients with CKD undergoing RYGB, VSG and gastric banding using BSA unadjusted iothalamate measured GFR and which also found stabilisation of measured GFR [11]. Interestingly, in the same study, measured GFR increased when adjustment for BSA was made. Similar findings have been observed when GFR was measured in patients with preserved renal function undergoing obesity surgery [4].

In our study stabilisation in renal function was also demonstrated using methodologies that incorporate direct measurement of cystatin C, such as CKD Epi cystatin creatinine. Cystatin C levels are not related to body or muscle mass and because EDTA GFR can be cumbersome and resource demanding, measurement of Cystatin C may be a useful alternative from a practical perspective.

Our study was limited by the small sample size, the heterogeneity of the aetiology of renal disease at baseline and type of operation used. Definitive interrogation of the effects of obesity surgery on measured GFR needs to be assessed in a prospective study with less aetiological heterogeneity, on type of operation used and longer follow up, as it can take years before any changes of GFR become evident.

## Conclusions

In conclusion we found stabilisation in renal function as measured by ^51^Cr-EDTA GFR or Cystatin C related assessments in patients with CKD 12 months after obesity surgery. Until further data emerges, where the surgical risk is high, it is critical to balance the risk and benefits of obesity surgery, especially if renal function may not improve as often as previously suggested.

## Abbreviations

BMI: Body mass index
CKD Epi: Chronic kidney disease epidemiology collaboration
EDTA: Ethylenediamine tetraacetic acid
eGFR: Estimated glomerular filtration rate
MDRD: Modification of diet in renal disease
